# Drugs of Abuse Can Entrain Circadian Rhythms

**DOI:** 10.1100/tsw.2007.234

**Published:** 2007-11-02

**Authors:** Ann E. K. Kosobud, Andrea G. Gillman, Joseph K. Leffel, Norman C. Pecoraro, G. V. Rebec, William Timberlake

**Affiliations:** ^1^ Department of Neurology, Indiana University School of Medicine, 975 W. Walnut St., IB 455, Indianapolis, IN 46202, USA; ^2^ Department of Psychological and Brain Sciences, Indiana University, 1101 E. 10^th^ St., Bloomington, IN 47405, USA; ^3^ Department of Physiology, University of California, 13 Parnassus Ave., San Francisco, CA 94143-0444, USA

**Keywords:** circadian rhythm, drug abuse, addiction, methamphetamine, fentanyl, nicotine, alcoholism, haloperidol, feeding, entrainment

## Abstract

Circadian rhythms prepare organisms for predictable events during the Earth's 24-h day. These rhythms are entrained by a variety of stimuli. Light is the most ubiquitous and best known zeitgeber, but a number of others have been identified, including food, social cues, locomotor activity, and, most recently drugs of abuse. Given the diversity of zeitgebers, it is probably not surprising that genes capable of clock functions are located throughout almost all organs and tissues. Recent evidence suggests that drugs of abuse can directly entrain some circadian rhythms. We have report here that entrainment by drugs of abuse is independent of the suprachiasmatic nucleus and the light/dark cycle, is not dependent on direct locomotor stimulation, and is shared by a variety of classes of drugs of abuse. We suggest that drug-entrained rhythms reflect variations in underlying neurophysiological states. This could be the basis for known daily variations in drug metabolism, tolerance, and sensitivity to drug reward. These rhythms could also take the form of daily periods of increased motivation to seek and take drugs, and thus contribute to abuse, addiction and relapse.

## INTRODUCTION: CHARACTERISTICS OF CIRCADIAN RHYTHMS

It is well known that most living organisms use circadian timing systems to synchronize their overall rest-activity rhythm with the cycle of day and night produced by the Earth's rotation. In vertebrates, critical control circuitry for the rest-activity cycle is located in the suprachiasmatic nucleus (SCN)[59]. This nucleus, tied directly to special light-sensitive ganglion cell receptors in the vertebrate eye, responds to light-dark transitions in the 24-h range by regulating and integrating a large number of physiological processes that produce and modify the basic rest-activity rhythm. The characteristic period of the restactivity rhythm has been tied experimentally to specific gene loci in mice, hamsters, and fruit flies[22,35,51,56].

The relatively recent advent of high-speed travel by humans across multiple time zones has helped to make clear that the circadian rest-activity cycle is entrained and "self-organized", rather than directly elicited and defined by transitions between light and dark. For example, removing light-dark transitions (in the absence of potential substitute cues) produces a free-running rhythm with a timing that differs slightly from the circadian[41]. The result is a self-organized rhythm that shifts its onset and offset time slowly around the 24-h clock. In another example, rapidly crossing multiple time zones (thereby changing the relative timing of existing light-dark transitions) reveals the ability of the rest-activity rhythm to reset its onset by moving a few hours each day until it matches the new light-dark transitions[41]. The integrated qualities of the circadian rest-activity rhythm are also revealed by multiple physiological and behavioral changes which begin 1.5–3 h before, and in apparent anticipation of, the entraining light-dark transitions.

It has become clear that light-dark transitions are not the only zeitgebers (entrainers of circadian rhythms). For example, the rest-activity cycle can be entrained by light pulses (so-called skeleton photo periods)[52,53], by sounds[37], and by social cues, including maternal signals or presence of a conspecific[40,44]. Circadian rhythms of anticipatory behavior can be entrained by the daily availability of a sizeable meal for a hungry animal[6,39]. Not only can the circadian feeding rhythm appear independent of a free-running circadian rest-activity cycle, but the two rhythms involve different neurophysiological substrates. The circadian rest-activity rhythm entrained by the light-dark cycle depends on an intact SCN, but the food-entrained rhythm does not[8]. After many incomplete leads, recent research has implicated the dorsomedial nucleus of the hypothalamus in the timing and expression of food-entrainable circadian rhythms[20,38].

In short, most organisms have evolved the ability to time and anticipate survival-related events occurring at a near-circadian interval. These circadian rhythms can be exquisitely sensitive to the type of cue (zeitgeber) evolved to entrain them, and insensitive to the presentation of other stimuli. They have a limited range of entrainment, so that zeitgebers scheduled at intervals much smaller or larger than 24 h fail to entrain circadian rhythms. The effective range varies somewhat between zeitgebers, individuals, and conditions, but is in the neighborhood of 21–30 h. Circadian rhythms tend to be self-sustaining in the absence of their zeitgeber(s), almost perpetually so in the case of the sleep-wake rhythm, and, for at least a few days, in the case of anticipatory wheel running entrained by meals[11,15,63]. Behavioral expressions that have disappeared can re-emerge after a single presentation of their zeitgeber or changes in the underlying motivating conditions. For example, meal-entrained locomotor rhythms apparently eliminated by *ad libitum* feeding can re-emerge after a single day of deprivation[7,10,62]. Some rhythms wax and wane independent of fulfillment; for example, during sleep deprivation, predictable periods of increased and decreased sleep latency can be measured[73]; similarly a fasting person will experience cycles of increasing then decreasing hunger.

## DRUGS OF ABUSE INTERACT WITH CIRCADIAN RHYTHMS

### Drugs Modify Characteristics of Circadian Rhythms

Considerable anecdotal evidence suggests the interaction of circadian rhythms and drug abuse. For example, drug addicts are commonly reported to show disrupted sleep, activity cycles, and eating habits. Researchers traditionally have assumed that these effects were indirect, mediated by drug-produced changes in personality characteristics, motivation, and levels of activity or fatigue. However, recent data provide evidence that addictive drugs can directly affect ongoing circadian rhythms. For example, cocaine alters the circadian rhythm of autonomic and immune mechanisms[28]. Chronic cocaine use appears to impair sleep, and this impairment worsens in the first few weeks of abstinence[42]. A single dose of 3,4-methylenedioxymethamphetamine (MDMA; ecstasy) can cause long-term changes in sleep and motor activity patterns[5]. Similarly, administration of ethanol[3,14], morphine[33], and phenobarbital[21] alters the circadian patterns of body temperature and activity. Finally, chronic methamphetamine (MA) can restore circadian locomotor rhythms in SCN-lesioned rats[26], and uncouple locomotor rhythms from the SCN in intact rats[34,57,58]. Interestingly, these MA-induced rhythms are food entrainable, suggesting that food- and drug-entrained rhythms share some common substrate[27].

### Circadian Activity Rhythms also Modify the Effects of Drugs

The actions of many therapeutic drugs are known to be affected by the circadian rest-activity cycle. For some drugs used to treat hypertension and cancer, time of day that the drug is taken has a critical effect on efficacy and safety of the drug[23,24,46,47,60,68]. Thus, it is not surprising that the rest-activity cycle also modifies the effects of drugs of abuse. For example, problems associated with drug abuse (withdrawal, unusual reaction, overdose) show a circadian pattern, with ER visits concentrating around late afternoon/early evening[16,43,55]. The phase of the light-dark cycle affects ethanol hypothermia[3], and sensitization to methylphenidate[19] and cocaine[4].

Further, studies with genetically altered mice have shown that the circadian clock genes influence the actions of abused drugs. Compared to their wild-type controls, mice lacking the *clock* gene show greater preference for an environment paired with cocaine; they also have a higher basal firing rate and bursting in neurons from the ventral tegmental area, a midbrain nucleus implicated in drug reward[36]. The *Per* gene plays a role in regulating behavioral responses to cocaine[1] and ethanol[61,70,71]. Further, circadian rhythms occur in neurotransmitter systems implicated in actions of drugs of abuse[9,29,30,31,45].

Finally, voluntary drug intake is affected by circadian rhythms. Rats subjected to multiple phase shifts in the light-dark cycle increased their intake of ethanol[18]. Ethanol intake is generally higher in the active phase[17], although the pattern of intake is strain dependent[2,25,48]. Also, rats self-administering cocaine i.v. appear to be most sensitive to the reinforcing effects of cocaine when it is available in the middle of the light or dark period, and less sensitive 1 h after the transition to light or dark[4]. Intracranial self-stimulation also occurs mostly in the active phase[54,64].

## DRUGS OF ABUSE CAN DIRECTLY ENTRAIN CIRCADIAN ACTIVITY RHYTHMS

In addition to interactions between circadian rhythms and drugs of abuse, there is increasing evidence that drugs of abuse directly entrain anticipatory circadian activity, in a manner not unlike that produced by established zeitgebers of light-dark transitions, meals, locomotor activity, and social cues. The strongest evidence that drugs can serve as zeitgebers is that drugs administered at 24-h intervals, whether by injection or voluntary intake, reliably produce circadian anticipatory activity that precedes daily drug availability by an hour or more, and persists for several days when the drug is withdrawn. For example, daily injections of MA rapidly result in an elevation of circadian locomotor activity preceding the injection, an elevation that persists for 2 days after injections stop[57,58].

To eliminate possible confounding of drug entrainment effects with the activity effects of light-dark transitions or uncontrolled ingestion of large meals at a particular time of day, we tested the effects of daily MA injections under conditions of constant low illumination with feeding rate limited to a maximum of two 95-mg pellets within any 5-min. interval. Fig. 1 shows average wheel turns in 1-h bins for rats given MA injections at 24 h. It can be seen that wheel turns increased in hours 22 and 23, immediately preceding the unsignaled circadian drug exposure.

After the last MA injection in this series, the rats were left undisturbed for 48 h. As expected, anticipatory wheel running was observed starting at about hour 23. There was no drug injection or any other cue at hour 24, and wheel running activity remained elevated for several hours during the time of the previous direct effects of drugs on behavior[32]. This persistent locomotor activity following the daily

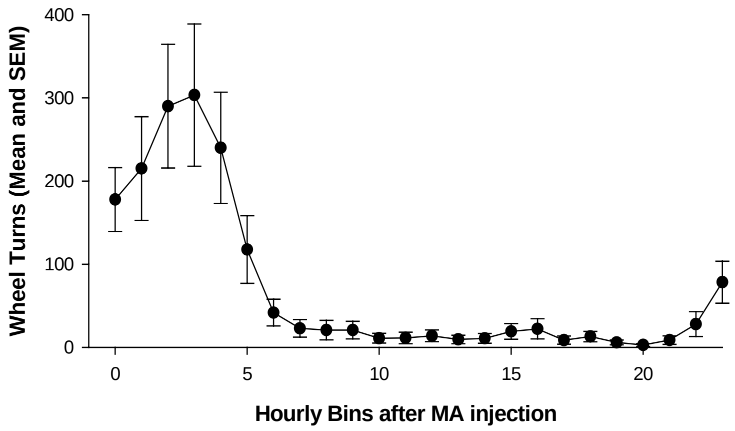

**FIGURE 1**. Wheel running (Mean ± SEM) in eight rats given MA (2 mg/kg) injections at 24-h intervals. Four of the rats were given ten injections at time 1200; the other four rats were given 17 injections at time 1900. In the figure, the injection time is normalized to time 0. MA had a direct stimulant effect that lasted about 5 h. Circadian anticipatory wheel running can be seen beginning at about hour 22, 2 h before the next injection. These rats were housed without time-of-day cues, under constant dim light, and rate-limited feeding. (Modified from Kosobud et al.[32]; used with permission.)

drug injection time, but in the absence of the drug injection, suggested the possibility that the locomotor activity previously elicited by the drug might have acted as a circadian zeitgeber, entraining one or both of the pre- and postinjection running activity. To test this possibility, we again gave rats a series of MA injections spaced at 24-h intervals, but this time we confined them to a small cage within the circadian chamber for 5 h immediately following the drug administrations to prevent gross locomotor activity. The same rats were then given a second series of 20 daily injections at a different time, while allowing free access to the wheel. Anticipatory wheel running developed normally in both cases and did not appear to differ in amount (Fig. 2). Thus, intense bouts of postdrug gross motor behavior do not appear to be required for the entrainment or expression of anticipatory drug-related circadian rhythms, although some combination of gross motor behavior and its accompaniments appears to affect rhythms following drug injection.

## THE HYPOTHESIS THAT DRUGS OF ABUSE FORM A CLASS OF CIRCADIAN ENTRAINING STIMULI THAT SHARE FUNCTIONAL CONSEQUENCES FOR DRUG ABUSE AND ADDICTION

Given that both cocaine and MA entrain circadian activity patterns[67], a question of practical and theoretical importance is whether other drugs of abuse also entrain circadian rhythms, and the extent to which entrainment is linked to their addictive properties. To empirically address whether entrainment is an important property of abused drugs, we are engaged in a program of screening drugs from a variety of classes, including fentanyl (an opiate), nicotine, ethanol, MA, and two nonabused drugs as controls, p-OH amphetamine (which has peripheral sympathomimetic actions like MA and cocaine, but does not cross the blood brain barrier), and haloperidol, which blocks DA receptors.

Our preliminary results to date indicate that nicotine and fentanyl given on 24-h schedules also entrain anticipatory activity, while haloperidol and a saline injection control group do not[74,75]. Further, Fig. 3 shows average standardized wheel turns for rats given access to a 10% alcohol/water solution for 1 h daily. Wheel turns were summed into 1-h bins; time 0 is the hour during which alcohol was available. Elevated wheel running can be observed in the last hour prior to the next

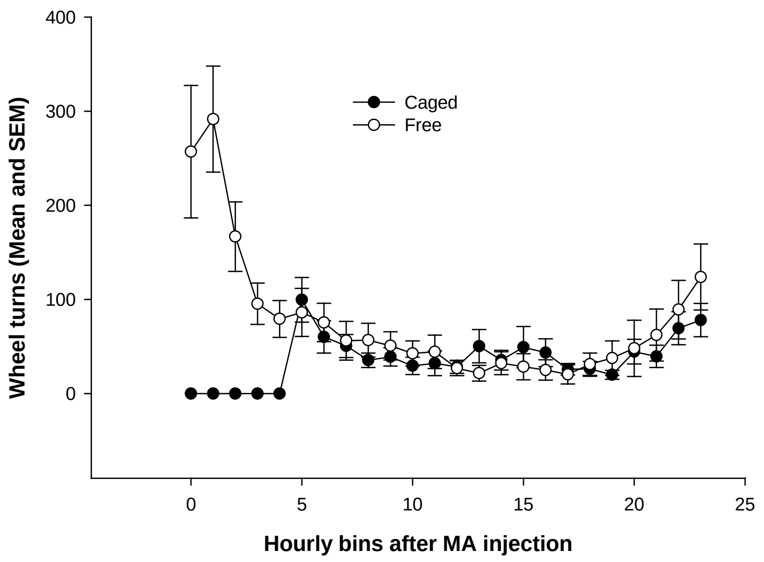

**FIGURE 2.** Wheel running (Mean ± SEM) in seven rats given injections of MA (2 mg/kg) at 24-h intervals. Following a series of 26 saline injections (data not shown), rats were given 20 injections of MA and then confined to small cages for 5 h, to prevent the sustained intense wheel running that normally follows MA administration. Following the Caged phase, the rats were given 16 more injections of MA and allowed free access to the running wheel (phase Free). Data for each subject were smoothed using a 1-h moving average, summed into 1-h bins, and averaged across animals. The rats were housed without time-of-day cues, under constant dim lighting, and with rate-limited feeding. MA was administered at time 0, and both groups showed anticipatory wheel running at hours 22 and 23. This result suggests that the locomotor activity directly stimulated by MA is not required for circadian anticipation.

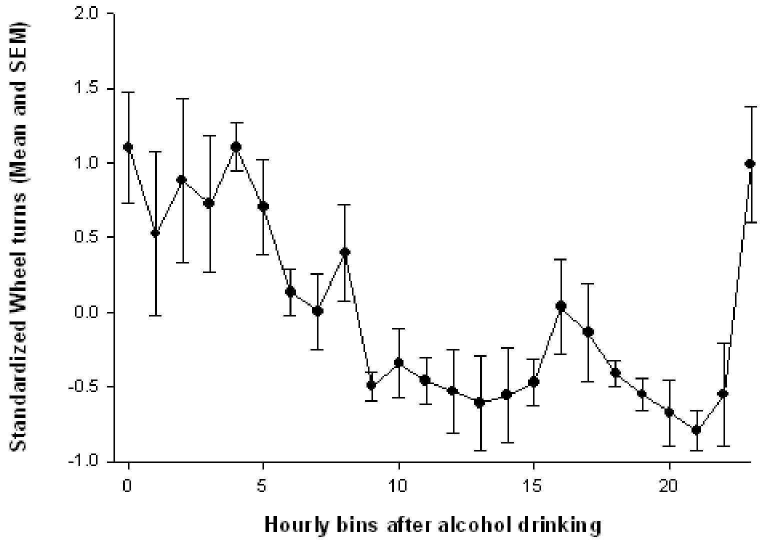

**FIGURE 3**. Standardized wheel turns (Mean ± SEM) for eight rats given 1-h access to a 10% ethanol/water solution at 24-h intervals on 25 days. This study used HAD (High Alcohol Drinking) rats, selectively bred for high intake of ethanol. Access was given during hour 0, and stimulated wheel running for 5–6 during and after the drinking period. The rats were housed without time-of-day cues, under constant dim lighting, and with rate-limited feeding. Circadian anticipatory wheel running can be seen at hour 23, 1 h before the next access period[72].

(uncued) alcohol session. It is worth pointing out that these rats were obtaining only 3–4 kCal/day from alcohol, compared with a total calorie consumption of 30–40 kCal per day (approximately 10% of daily calories). This level is well below the threshold for meal entrainment, which has been estimated at about 30% of daily calories, or about 8 g (24 kCal) of standard lab chow pellets[65].

An important next step is to determine common factors that may underlie these results for different drugs. For example, many, if not all, drugs of abuse share an ability to increase dopaminergic transmission in the mesolimbic dopamine (DA) system[12]. However, these drugs also influence this system through different primary mechanisms, and some drugs have additional actions that contribute to their abuse potential. Thus, cocaine and MA act directly at dopaminergic synapses to enhance transmission. Nicotine and opiates stimulate DA neuron firing at nicotinic acetylcholine and μ-opiate receptors, respectively. Finally, although the direct actions of ethanol are inhibitory (enhancing GABA-mediated Cl- flux, inhibiting NMDA transmission), it has been shown to excite DA neurons *in vivo*[13].

## A SECOND FORM OF TESTING THE HYPOTHESIS THAT DRUGS FORM A CLASS OF CIRCADIAN ENTRAINING STIMULI: THE EFFECTS OF INFRADIAN (LONG TAU) CYCLES OF DRUG ADMINISTRATION

When a zeitgeber of light-dark transitions is given at an interval that well exceeds the typical circadian entrainment range (e.g., 29 or 31 h between transitions), the circadian rest-activity cycle fails to entrain to this “day” length, and the rhythms start to free-run. To investigate whether this effect might apply to meal anticipation, White and Timberlake[66] presented a large meal on a 31-h schedule and obtained similar results. Anticipatory wheel running did not occur just before the 31-h schedule, but instead occurred approximately 24 h after the last large feeding. White and Timberlake[66] called this burst “ensuing activity” because although it occurred at approximately a circadian interval, it was not anticipatory in any functional sense. As in the case of a 24-h meal-entrained anticipatory activity burst, the circadian-ensuing activity showed damped oscillation at an approximately 24-h interval for several days following the final meal. This result was also present in a group of SCN-lesioned rats.

The question we are addressing with these tests is whether drug-related circadian anticipatory rhythms on such presentation schedules show the same effects as meals. If similar circadian mechanisms are involved, we predict that animals exposed to a 31-h cycle of drug administration should show elevated activity at an interval closer to 24 h than to 31 h, and that this cycle would be reset with each administration. It is worth noting that testing the effect of a 31-h meal schedule on entrainment was complicated by the need, at least in rats, to offer a supplementary meal to maintain a stable body weight. Thus, White and Timberlake[66] offered their rats a second, significantly smaller meal, approximately 6 h after the 31-h meal. The size of this meal appeared to fall below the threshold for producing notable ensuing activity.

Fortunately, testing the effect of a 31-h schedule of addictive drug administration can be done with a single injection at each 31 h because the drug is not necessary for tissue-need based survival. Thus, we can assess more cleanly whether anticipatory drug activity is based on a circadian cycle. Thus, White[67], using cocaine injections, showed that ensuing activity also follows cocaine injections by a near-circadian interval despite there never being injections at the 24-h interval. Similarly, we have shown (see Fig. 4) that circadian-like ensuing activity also follows MA injections given on a 31-h schedule in both intact and SCN-lesioned rats[49,50].

## SUMMARY AND CONCLUSIONS

We and other researchers have compiled evidence strongly supporting the conclusion that drugs of abuse can act as zeitgebers by entraining circadian anticipatory activity rhythms. This entrainment does not appear to require the presence of a light-dark cycle, a 24-h feeding rhythm, or an intact SCN (the suprachiasmatic

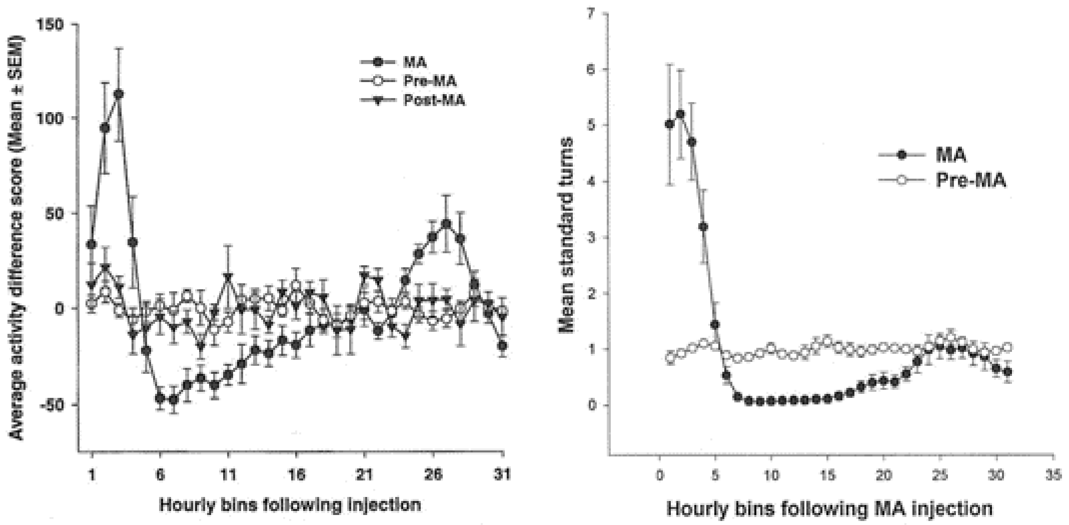

**FIGURE 4.** Wheel running in eight rats with (right panel) and eight rats without (left panel) SCN lesions, given 24 injections of MA (2 mg/kg) at 31-h intervals. The rats were housed without time-of-day cues, under constant dim lighting, and with rate-limited feeding. MA was administered at time 0. With a 31-h schedule, no anticipatory wheel running develops. Instead, a transient increase in wheel running occurs 24–27 h after each injection. The left panel shows the pattern of activity in normal rats given MA, as well as during a pre- and post-MA phase when they received no injections[49; used with permission]. The right panel shows data for rats in which the SCN had been lesioned[50]. The lesion eliminated the rest-activity cycle in these rats, but they still showed circadian ensuing activity.

nucleus, assumed by most investigators to be a master clock of circadian and ultradian rhythms). The resemblance of the behavioral effects of 24-h administration of addictive drugs to the behavioral effects of 24-h administration of food suggests similarities in the underlying neurophysiological mechanisms. It is worth noting that the incorporation of meals and of abused drugs both present a challenge to homeostasis, and therefore to the degree that these events can be accurately predicted, appropriate compensatory mechanisms would be expected to be called into play[69]. The circadian locomotor activity that precedes daily drug administration may be an index of the seeking and compensatory reactions that make up drug addiction. It remains an open question the extent to which the apparent similarities between meal- and drug-entrained rhythms arises through shared peripheral and central mechanisms.

Research in the last decade has shown that daily (circadian) injections of MA and cocaine, and daily access to alcohol, entrain anticipatory circadian rhythms; preliminary findings suggest that nicotine and fentanyl also entrain such rhythms, while haloperidol and saline do not. These circadian anticipatory rhythms are revealed by a pattern of locomotor activity that begins to increase a few hours prior to the next drug administration. After the final drug administration in a series, periods of increased activity continue to appear at about 24-h intervals for several days. A similar pattern of increasing, then declining wheel running activity is observed about 24 h following injections given on a 31-h interval. To date, all drugs that entrain anticipation appear to also evoke this circadian ensuing activity, but we do not know whether similar or identical neural and physiological mechanisms are in play.

In the case of MA injections, this drug-entrained anticipatory circadian activity does not require the presence of an intact SCN, does not depend on expression of the direct locomotor stimulant effects of the drug, and is unaffected by the presence or absence of food-entrained circadian rhythms. We suggest that drug-entrained rhythms reflect variations in underlying neurophysiological states that alter an organism's responses to the drug. We are currently investigating whether circadian scheduling enhances intake and motivation to seek drugs. Future studies of the mechanisms underlying these rhythms will add to our understanding of the actions of drugs of abuse, and how episodes of drug taking evolve into chronic abuse and addiction.

## ACKNOWLEDGMENTS

Supported by grants from NIDA (R01 DA01692 and R21 DA11092), NIAAA (P50 AA07611), and NIMH (37892).

## References

1. Abarca, C., Albrecht, U., and Spanagel, R. (2002) Cocaine sensitization and reward are under the influence of circadian genes and rhythm. *Proc. Natl. Acad. Sci. U. S. A.* **99,** 9026–9030.
2. Agabio, R., Cortis, G., Fadda, F., Gessa, G.L., Lobina, C., Reali, R., and Colombo, G. (1996) Circadian drinking pattern of Sardinian alcohol-preferring rats. *Alcohol Alcohol.* **31,** 385–388.
3. Baird, T.J., Briscoe, R.J., Vallett, M., Vanecek, S.A., Holloway, F.A., and Gauvin, D.V. (1998) Phase-response curve for ethanol: alterations in circadian rhythms of temperature and activity in rats. *Pharmacol. Biochem. Behav.* **61,** 303–315.
4. Baird, T.J. and Gauvin, D. (2000) Characterization of cocaine self-administration and pharmacokinetics as a function of time of day in the rat. *Pharmacol. Biochem. Behav.* **65,** 289–299.
5. Balogh, B., Molnar, E., Jakus, R., Quate, L., Olverman, H.J., Kelly, P.A., Kantor, S., and Bagdy, G. (2004) Effects of a single dose of 3,4-methylenedioxymethamphetamine on circadian patterns, motor activity and sleep in drug-naive rats and rats previously exposed to MDMA. *Psychopharmacology (Berl.)* **173,** 296–309.
6. Bolles, R.C. and De Lorge, J. (1962) The rat's adjustment to a-diurnal feeding cycles. *J. Comp. Physiol. Psychol.* **55,** 760–762.
7. Bolles, R.C. and Moot, S.A. (1973) The rat's anticipation of two meals a day. *J. Comp. Physiol. Psychol.* **83,** 510–514.
8. Boulos, Z., Rosenwasser, A.M., and Terman, M. (1980) Feeding schedules and the circadian organization of behavior in the rat. *Behav. Brain Res.* **1,** 39–65.
9. Bruinink, A., Lichtensteiger, W., and Schlumpf, M. (1983) Ontogeny of diurnal rhythms of central dopamine, serotonin and spirodecanone binding sites and of motor activity in the rat. *Life Sci.* **33,** 31–38.
10. Clarke, J.D. and Coleman, G.J. (1986) Persistent meal-associated rhythms in SCN-lesioned rats. *Physiol. Behav.* **36,** 105–113.
11. Coleman, G.J., Harper, S., Clarke, J.D., and Armstrong, S. (1982) Evidence for a separate meal-associated oscillator in the rat. *Physiol. Behav.* **29,** 107–115.
12. Di Chiara, G. (1995) The role of dopamine in drug abuse viewed from the perspective of its role in motivation. *Drug Alcohol Depend.* **38,** 95–137. Erratum in *Drug Alcohol Depend.* **39(2),** 155.
13. Diana, M., Rossetti, Z.L., and Gessa, G. (1993) Rewarding and aversive effects of ethanol: interplay of GABA, glutamate and dopamine. *Alcohol Alcohol. Suppl.* **2,** 315–319.
14. Eastman, C.I., Stewart, K.T., and Weed, M.R. (1994) Evening alcohol consumption alters the circadian rhythm of body temperature. *Chronobiol. Int.* **11,** 141–142.
15. Edmonds, S.C. and Adler, N.T. (1977) The multiplicity of biological oscillators in the control of circadian running activity in the rat. *Physiol. Behav.* **18,** 921–930.
16. Erickson, T.B., Lee, J., Zautcke, J.L., and Morris, R. (1998) Analysis of cocaine chronotoxicology in an urban ED. *Am. J. Emerg. Med.* **16,** 568–571.
17. Files, F.J., Lewis, R.S., and Samson, H.H. (1993) Ethanol self-administration by alcohol-nonpreferring (NP) rats in a continuous access situation. *Alcohol* **10,** 139–144.
18. Gauvin, D.V., Baird, T.J., Vanecek, S.A., Briscoe, R.J., Vallett, M., and Holloway, F.A. (1997) Effects of time-of-day and photoperiod phase shifts on voluntary ethanol consumption in rats. *Alcohol. Clin. Exp. Res.* **21,** 817–825.
19. Gaytan, O., Yang, P., Swann, A., and Dafny, N. (2000) Diurnal differences in sensitization to methylphenidate. *Brain Res.* **864,** 24–39.
20. Gooley, J.J., Schomer, A., and Saper, C.B. (2006) The dorsomedial hypothalamic nucleus is critical for the expression of food-entrainable circadian rhythms. *Nat. Neurosci.* **9,** 398–407.
21. Groh, K.R., Ehret, C.F., Peraino, C., Meinert, J.C., and Readey, M.A. (1988) Circadian manifestations of barbiturate habituation, addiction and withdrawal in the rat. *Chronobiol. Int.* **5,** 153–166.
22. Grosse, J., Loudon, A.S., and Hastings, M.H. (1995) Behavioural and cellular responses to light of the circadian system of tau mutant and wild-type Syrian hamsters. *Neuroscience* **65,** 587–597.
23. Halberg, F., Cornelissen, G., Ulmer, W., Blank, M., Hrushesky, W., Wood, P., Singh, R.K., and Wang, Z. (2006) Cancer chronomics. III. Chronomics for cancer, aging, melatonin and experimental therapeutics researchers. *J. Exp. Ther. Oncol.* **6,** 73–84.
24. Hermida, R.C., Ayala, D.E., Smolensky, M.H., and Portaluppi, F. (2007) Chronotherapy in hypertensive patients: administration-time dependent effects of treatment on blood pressure regulation. *Expert Rev. Cardiovasc. Ther.* **5,** 463–475.
25. Hiller-Sturmhofel, S. and Kulkosky, P. (2001) Chronobiological regulation of alcohol intake. *Alcohol Res. Health* **25,**

141–148.

26. Honma, K., Honma, S., and Hiroshige, T. (1987) Activity rhythms in the circadian domain appear in suprachiasmatic nuclei lesioned rats given methamphetamine. *Physiol. Behav.* **40,** 767–774.
27. Honma, S., Honma, K.I., and Hiroshige, T. (1989) Methamphetamine induced locomotor rhythm entrains to restricted daily feeding in SCN-lesioned rats. *Physiol. Behav.* **45,** 1057–1065.
28. Irwin, M.R., Olmos, L., Wang, M., Valladares, E.M., Motivala, S.J., Fong, T., Newton, T., Butch, A., Olmstead, R., and Cole, S.W. (2007) Cocaine dependence and acute cocaine induce decreases of monocyte proinflammatory cytokine expression across the diurnal period: autonomic mechanisms. *J. Pharmacol. Exp. Ther.* **320,** 507–515.
29. Kafka, M.S., Marangos, P.J., and Moore, R.Y. (1985) Suprachiasmatic nucleus ablation abolishes circadian rhythms in rat brain neurotransmitter receptors. *Brain Res*. **327,** 344–347.
30. Kafka, M.S., Wirz-Justice, A., and Naber, D. (1981) Circadian and seasonal rhythms in alpha- and beta-adrenergic receptors in the rat brain. *Brain Res.* **207,** 409–419.
31. Kafka, M.S., Wirz-Justice, A., Naber, D., and Wehr, T.A. (1981) Circadian acetylcholine receptor rhythm in rat brain and its modification by imipramine. *Neuropharmacology* **20,** 421–425.
32. Kosobud, A.E., Pecoraro, N.C., Rebec, G.V., and Timberlake, W. (1998) Circadian activity precedes daily methamphetamine injections in the rat. *Neurosci. Lett.* **250,** 99–102.
33. Marchant, E.G. and Mistlberger, R.E. (1995) Morphine phase-shifts circadian rhythms in mice: role of behavioural activation. *Neuroreport* **7,** 209–212.
34. Masubuchi, S., Honma, S., Abe, H., Ishizaki, K., Namihira, M., Ikeda, M., and Honma, K. (2000) Clock genes outside the suprachiasmatic nucleus involved in manifestation of locomotor activity rhythm in rats. *Eur. J. Neurosci.* **12,** 4206–4214.
35. Matsumoto, A., Motoshige, T., Murata, T., Tomioka, K., Tanimura, T., and Chiba, Y. (1994) Chronobiological analysis of a new clock mutant, Toki, in Drosophila melanogaster. *J. Neurogenet.* **9,** 141–155.
36. McClung, C.A., Sidiropoulou, K., Vitaterna, M., Takahashi, J.S., White, F.J., Cooper, D.C., and Nestler, E.J. (2005) Regulation of dopaminergic transmission and cocaine reward by the Clock gene. *Proc. Natl. Acad. Sci. U. S. A.* **102,** 9377–9381.
37. Menaker, M. and Eskin, A. (1966) Entrainment of circadian rhythms by sound in Passer domesticus. *Science* **154,** 1579–1581.
38. Mieda, M., Williams, S.C., Richardson, J.A., Tanaka, K., and Yanagisawa, M. (2006) The dorsomedial hypothalamic nucleus as a putative food-entrainable circadian pacemaker. *Proc. Natl. Acad. Sci. U. S. A.* **103,** 12150–12155.
39. Mistlberger, R.E. and Rechtschaffen, A. (1984) Recovery of anticipatory activity to restricted feeding in rats with ventromedial hypothalamic lesions. *Physiol. Behav.* **33,** 227–235.
40. Mistlberger, R.E. and Skene, D.J. (2004) Social influences on mammalian circadian rhythms: animal and human studies. *Biol. Rev. Camb. Philos. Soc.* **79,** 533–556.
41. Moore-Ede, M.C., Sulzman, F.M., and Fuller, C.A. (1982) *The Clocks that Time Us*. Harvard University Press, Cambridge, MA.
42. Morgan, P.T., Pace-Schott, E.F., Sahul, Z.H., Coric, V., Stickgold, R., and Malison, R.T. (2006) Sleep, sleep-dependent procedural learning and vigilance in chronic cocaine users: evidence for occult insomnia. *Drug Alcohol Depend.* **82,** 238–249.
43. Morris, R.W. (1987) Circadian and circannual rhythms of emergency room drug-overdose admissions. *Prog. Clin. Biol. Res.* **227B,** 451–457.
44. Mrosovsky, N., Reebs, S.G., Honrado, G.I., and Salmon, P.A. (1989) Behavioural entrainment of circadian rhythms. *Experientia* **45,** 696–702.
45. Naber, D., Wirz-Justice, A., and Kafka, M.S. (1981) Circadian rhythm in rat brain opiate receptor. *Neurosci. Lett.* **21,** 45–50.
46. Ohdo, S. (2007) Chronopharmacology focused on biological clock. *Drug Metab. Pharmacokinet.* **22,** 3–14.
47. Ohdo, S. (2007) Circadian rhythms in the CNS and peripheral clock disorders: chronopharmacological findings on antitumor drugs. *J. Pharmacol. Sci.* **103,** 155–158.
48. Pasley, J.N., Powell, E.W., and Halberg, F. (1987) Strain differences in circadian drinking behaviors of ethanol and water in rats. *Prog. Clin. Biol. Res.* **227B,** 467–471.
49. Pecoraro, N., Kosobud, A.E., Rebec, G.V., and Timberlake, W. (2000) Long T methamphetamine schedules produce circadian ensuing drug activity in rats. *Physiol. Behav.* **71,** 95–106.
50. Pecoraro, N., Kosobud, A.E.K., Rebec, G.V., and Timberlake, W. (2000) Methamphetamine promotes locomotor activity following a circadian interval in SCN-lesioned rats. Society for Neuroscience Abstracts (26), http://sfn.scholarone.com/itin2000/
51. Pickard, G.E., Sollars, P.J., Rinchik, E.M., Nolan, P.M., and Bucan, M. (1995) Mutagenesis and behavioral screening for altered circadian activity identifies the mouse mutant, Wheels. *Brain Res.* **705,** 255–266.
52. Pittendrigh, C.S. and Dann, S. (1976) A functional analysis of circadian pacemakers in nocturnal rodents. IV. Entrainment: pacemaker as clock. *J. Comp. Physiol.* **106**, 291–331.
53. Pittendrigh, C.S. and Minis, D.H. (1964) The entrainment of circadian oscillations by light and their role as photoperiodic clocks. *Am. Nat.* **98,** 261–294.
54. Prescott, R.G. (1967) Diurnal activity cycles and intracranial self-stimulation in the rat. *J. Comp. Physiol. Psychol.*

**64,** 346–349.

55. Raymond, R.C., Warren, M., Morris, R.W., and Leikin, J.B. (1992) Periodicity of presentations of drugs of abuse and overdose in an emergency department. *J. Toxicol. Clin. Toxicol.* **30,** 467–478.
56. Schwartz, W.J. and Zimmerman, P. (1990) Circadian timekeeping in BALB/c and C57BL/6 inbred mouse strains. *J. Neurosci.* **10,** 3685–3694.
57. Shibata, S., Minamoto, Y., Ono, M., and Watanabe, S. (1994) Age-related impairment of food anticipatory locomotor activity in rats. *Physiol. Behav.* **55,** 875–878.
58. Shibata, S., Minamoto, Y., Ono, M., and Watanabe, S. (1994) Aging impairs methamphetamine-induced free-running and anticipatory locomotor activity rhythms in rats. *Neurosci. Lett.* **172,** 107–110.
59. Silver, R. and Schwartz, W.J. (2005) The suprachiasmatic nucleus is a functionally heterogeneous timekeeping organ. *Methods Enzymol.* **393,** 451–465.
60. Smolensky, M.H., Hermida, R.C., and Portaluppi, F. (2007) Comparison of the efficacy of morning versus evening administration of olmesartan in uncomplicated essential hypertension. *Chronobiol. Int.* **24,** 171–181.
61. Spanagel, R., Rosenwasser, A.M., Schumann, G., and Sarkar, D.K. (2005) Alcohol consumption and the body's biological clock. *Alcohol. Clin. Exp. Res.* **29,** 1550–1557.
62. Stephan, F.K., Swann, J.M., and Sisk, C.L. (1979) Anticipation of 24-hr feeding schedules in rats with lesions of the suprachiasmatic nucleus. *Behav. Neural Biol.* **25,** 346–363.
63. Stephan, F.K., Swann, J.M., and Sisk, C.L. (1979) Entrainment of circadian rhythms by feeding schedules in rats with suprachiasmatic lesions. *Behav. Neural Biol.* **25,** 545–554.
64. Terman, M. and Terman, J.S. (1970) Circadian rhythm of brain self-stimulation behavior. *Science* **168,** 1242–1244.
65. White, W., Schwartz, G.J., and Moran, T.H. (1999) Meal-synchronized CEA in rats: effects of meal size, intragastric feeding, and subdiaphragmatic vagotomy. *Am. J. Physiol.* **276,** R1276–R1288.
66. White, W. and Timberlake, W. (1999) Meal-engendered circadian-ensuing activity in rats. *Physiol. Behav.* **65,** 625–642.
67. White, W., Feldon, J., Heidbreder, C.A., and White, IM. (2000) Effects of administering cocaine at the same versus varying times of day on circadian activity patterns and sensitization in rats. *Behav. Neurosci.* **114,** 972–982.
68. Wood, P.A., Du-Quiton, J., You, S., and Hrushesky, W.J. (2006) Circadian clock coordinates cancer cell cycle progression, thymidylate synthase, and 5-fluorouracil therapeutic index. *Mol. Cancer Ther.* **5,** 2023–2033.
69. Woods, S.C. and Ramsay, D.S. (2000) Pavlovian influences over food and drug intake. *Behav. Brain Res.* **110,** 175–182.
70. Yuferov, V., Bart, G., and Kreek, M.J. (2005) Clock reset for alcoholism. *Nat. Med.* **11,** 23–24.
71. Yuferov, V., Butelman, E.R., and Kreek, M.J. (2005) Biological clock: biological clocks may modulate drug addiction. *Eur. J. Hum. Genet.* **13,** 1101–1103.
72. Kosobud, A.E.K., Leffel, J., Rebec, G.V., and Timberlake, W. (2002) Scheduled daily access to alcohol solutions alters circadian patterns of locomotion in HAD rats. *Alcoholism: Clinical and Experimental Research* **26(5)**, 61A.
73. Richardson, G. S. (2005). The human circadian system in normal and disordered sleep*. J Clin. Psychiatry* **66 Suppl. 9**, 3-9.
74. Gillman, A.G., Leffel, J.K., Kosobud, A E K., and Timberlake, W. (In preparation a). Fentanyl, but not haloperidol, produces circadian activity patterns when administered on 24-hr or 31-hr schedules.
75. Gillman, A.G., Leffel, J.K., Kosobud, A.E.K., and Timberlake, W. (In preparation b). Both nicotine and alcohol produce circadian activity patterns when administered on 24-hr or 31-hr schedules.
